# RNAi-Mediated Knockdown of Catalase Causes Cell Cycle Arrest in SL-1 Cells and Results in Low Survival Rate of *Spodoptera litura* (Fabricius)

**DOI:** 10.1371/journal.pone.0059527

**Published:** 2013-03-26

**Authors:** Haiming Zhao, Xin Yi, Zhen Hu, Meiying Hu, Shaohua Chen, Rizwan-ul-Haq Muhammad, Xiaolin Dong, Liang Gong

**Affiliations:** Laboratory of Insect Toxicology, Key Laboratory of Pesticide and Chemical Biology, Ministry of Education, South China Agricultural University, Guangzhou, People’s Republic of China; National Institute of Agronomic Research, France

## Abstract

Deregulated reactive oxygen species (ROS) production can lead to the disruption of structural and functional integrity of cells as a consequence of reactive interaction between ROS and various biological components. Catalase (CAT) is a common enzyme existing in nearly all organisms exposed to oxygen, which decomposes harmful hydrogen peroxide, into water and oxygen. In this study, the full length sequence that encodes CAT-like protein from *Spodoptera litura* named *siltCAT* (GenBank accession number: JQ_663444) was cloned and characterized. Amino acid sequence alignment showed *siltCAT* shared relatively high conservation with other insect, especially the conserved residues which defined heme and NADPH orientation. Expression pattern analysis showed that *siltCAT* mRNA was mainly expressed in the fat body, midgut, cuticle and malpighian tube, and as well as over last instar larvae, pupa and adult stages. RNA interference was used to silence CAT gene in SL-1 cells and the fourth-instar stage of *S. litura* larvae respectively. Our results provided evidence that CAT knockdown induced ROS generation, cell cycle arrest and apoptosis in SL-1 cells. It also confirmed the decrease in survival rate because of increased ROS production in experimental groups injected with double-stranded RNA of CAT (dsCAT). This study implied that ROS scavenging by CAT is important for *S. litura* survival.

## Introduction

Superoxide (O_2_^−^) and hydrogen peroxide (H_2_O_2_) are normal byproducts of oxygen metabolism in cells, which can adversely react with proteins, DNA, membrane lipids, and other cellular components, leading to a variety of cytotoxic effects [1]–[3]. These partially reduced metabolites of O_2_ are often referred to as “reactive oxygen species” (ROS) due to their high reactivity with other cellular molecules [6]_._ Oxidative stress may be broadly defined as an imbalance between oxidant production and the antioxidant capacity of the cell. Although oxidative stress is considered to promote cell death in response to a variety of patho-physiological signals, existing evidence suggested that organisms tend to be in a pro-oxidant state and efficient oxidative defense system is critical to prevent cellular damage insulted by ROS [4], [5].

Insects have evolved both enzymatic and nonenzymatic defense mechanisms to remove activated oxygen species. Enzymeatic system employs antioxidant enzymes such as superoxide dismutase (SOD), catalase (CAT), and glutathione peroxidase (GPX) to neutralize ROS, which is central to ROS decomposition [6], [7]. CAT is one of the key antioxidant enzymes belonging to SOD/CAT system that prevents the formation of the hydroxyl radical (HO•). SOD is responsible for catalyzing the dismutation of superoxide anion into H_2_O_2_ and oxygen, while CAT further reduces H_2_O_2_ to water and molecular oxygen [8]. Changes in the activities of antioxidant enzymes have been shown to correlate with the occurrence of oxidative stress in various systems [9]. In insect, antioxidant enzymes participate in regulation of oxidative stress and maintain normal metabolism. In general, the better ability of an organism to resist oxidative stress or repair damage is associated with extended lifespan, owing to the protection of cells against apoptosis [10]. Although many studies demonstrated that CAT relieves oxidative stresses to protect the cells against apoptosis, there is very little information on molecular mechanisms of ROS-scavenging to resist oxidative stress on *Spodoptera litura* (Fabricius) [11]–[13].

*S. litura* is an economically important polyphagous pest in China, India, and Japan, causing considerable economic loss to many vegetable and field crops [14]. In recent years its outbreaks have been more frequent in Asia, mainly due to insecticide resistance [15]. Most of the commonly used insecticides, especially pyrethroids and carbamates, have been shown to fail to provide adequate control. Therefore, it is urgent to seek for new ways of preventing this pest from biological perspective. As insects are deficient in a selenium-dependent GPX that is H_2_O_2_ scavenger present in other organisms, CAT has been considered to be the sole scavenger of H_2_O_2_ in insects [16]. CAT may be a candidate target of insecticides, due to its important role in regulating oxidative stress in insect species [17]. Developmental changes in the activity of CAT were reported for the European corn borer, *Ostrnia nubilalis*, the silkworm, *Bombyx mori* and the Elateridae, *Pyrearinus termitilluminans* [18]–[20]. However, compared to CATs in mammals and bacteria [21], little is known about insect CATs especially the biochemical properties of insect CAT and its effects on the apoptosis.

In this study, with the aim to achieve a new pest control approach, we cloned insect CAT gene in *S. litura* and examined its related functional capabilities. The full-length cDNA encoding a CAT (*siltCAT*) from *S. litura* was cloned and characterized by Reverse Transcription-Polymerase Chain Reaction (RT-PCR) and Rapid-Amplification of cDNA Ends (RACE) technique. Expression pattern in different developmental stages and tissues was analyzed by real-time PCR and semi-quantitative RT-PCR. In addition, RNA interference (RNAi), as well as dsRNA injections, was employed to further investigate the consequences of CAT gene knockdown *in vitro* and *in vivo*. These results showed that *siltCAT* had major impact on survival in fourth-instar larvae of *S. litura* and CAT knockdown could induce apoptosis in SL-1 cells. Collectively, our results demonstrated CAT is essential to the survival of *S. litura*, implying that targeting CAT and related antioxidant system could be a novel approach for insect pest control.

## Materials and Methods

### Ethics Statement

No specific permits were required for the described studies. No specific permissions were required for these locations. We confirm that the location is not privately-owned or protected in any way. We confirm that the studies did not involve endangered or protected species.

### Insects and Cell Culture

Adult female and male *S. litura*, were reared under crowded conditions at constant temperature (26±1°C), 85(±5) % relative humidity and constant day/night cycle (16 hphotoperiod). They were fed daily with fresh cabbage leaves. For the first generation of isolated insects, the eggs of crowded females were washed and separated. The next generations were obtained by using eggs of individually reared females that were placed separately after hatching. All used insects were synchronized on the day of their adult moult. Depending on the objective of the experiments, females and/or males were used.

*S. litura* cell line (SL-1) was obtained from the Institute of Entomology, Sun Yat-sen University in China. Cells were subcultured every 3 days by inoculating in a 25 cm^2^ plastic tissue culture flask with 5×10^5^ cells in a total volume of 3 ml antibiotic-free Grace’s insect medium (Sigma-Aldrich, Washington, USA) containing 5.0% foetal bovine serum albumin(Sigma), 0.3% yeast extract (Sigma) and 0.3% lactalbumin hydrolysate (Sigma).

### RNA Preparation and cDNA Synthesis

Total RNA from different tissue samples was extracted from *S. litura* using the E.Z.N.A.™ total RNA isolation system kit (Omega Bio-tech, Doraville, GA, USA) according to the manufacturer's instructions. First-strand cDNA was synthesized in a 20 µL reaction volume using the RevertAid™ First Strand cDNA Synthesis Kit (Fermentas, Canada) under the conditions recommended by the manufacturer. For subsequent degenerate PCR, cDNA was synthesized from 1 µg of female adult RNA with oligo (dT)_18_ primer (TaKaRa, DaLian, China).

### Cloning of *siltCAT* Gene and Sequencing

A pair of degenerate oligonucleotide primers were designed based on conserved amino acid sequences of *DrRFeSP* (NM_164426) and *BomCAT* (NP_001036912) (Table 1). A middle fragment of the conserved intermediate coding region was initially amplified using this pair of degenerate primers. PCR was performed with ExTaq polymerase (TaKaRa, DaLian, China) under the following condition: 94°C for 2 min, followed by 35 cycles at 94°C for 30 sec, 58°C for 1 min, 72°C for 1 min and finally 72°C for 10 min using the degenerate primer pair CAT-f and CAT-r. A BD SMART RACE cDNA amplification kit (BD Bioscience Clontech, CA, US) was used to obtain the full-length of *siltCAT*. Based on the initial results, specific primers for the 5′- and 3′- RACE were designed (Table 1). The specific primers 5CAT-1 and 5CAT-2 were used for 5′-RACE, while 3CAT-1 and 3CAT-2 were used for 3′-RACE (Table 1). In brief, using the 5′−/3′-RACE cDNAs as a template, PCR was performed using the 5CAT-1/3CAT-1 primer and Universal Primer Mix (UPM, Clontech) by denaturing at 94°C for 4 min, followed by 5 cycles at 94°C for 30 sec and 72°C for 3 min, then 5 cycles at 94°C for 30 sec, 70°C for 30 sec and 72°C for 3 min, followed by 25 cycles at 94°C for 30 sec, 68°C/55°C for 30 sec, and 72°C for 3 min. The PCR program was ended with a final extension step at 72°C for 10 min. Nested PCR was carried out with the first-round PCR product as a template and the Nested Universal Primer A (NUP, Clontech) and 5CAT-2/3CAT-2 primer. The reaction conditions consisted of the followings: 4 min of initial preheating at 94°C, 5 cycles at 94°C for 30 sec and 72°C for 3 min, then 5 cycles at 94°C for 30 sec, 70°C for 30 sec and 72°C for 3 min, followed by 25 cycles at 94°C for 30 sec, 65°C/55°C for 30 sec, and 72°C for 3 min. The PCR program was ended with a final extension step at 72°C for 10 min.

**Table 1:** Primers used in this study.

primers	Primer sequence
*Dgenerate primers*	
CAT-f	5′-CATGACATCACCMAGTAYWGTG-3′
CAT-r	5′-KRCSTCCATCMCGCTGG WAGTT-3′
*For RACE*	
5CAT-1	5′-CAACAGTGGAGAATCTCACAGC-3′
5CAT-2	5′- GTATGGATGAAACTAGGGAACA-3′
3CAT-1	5′-ATGACTATGGCTCAAGCGGAAA-3′
3CAT-2	5′-AGTTGAACAGATTGCATTCATG-3′
*For RT-PCR and RT-qPCR*	
CAT-qF	5′-CAAACTGTTGGCAAGAATGGA-3′
CAT-qR	5′-GCACAACACGCTCTGGAATG-3′
Actin-F	5′-GCCAACAGGGAGAAGATG-3′
Actin-R	5′-CGGTGGTGGTGAAAGAGTA-3′
*For siltCAT dsRNA synthesis*	
dsCAT-F	5′-CGTGGATTTGCTGTTAAATTC-3′
dsCAT-R	5′-TGACTATGGCTCAAGCGGAAAG-3′
*For GFP dsRNA synthesis*	
dsGFP-F	5′-AAGGGCGAGGAGCTGTTCACCG-3′
dsGFP-R	5′-CAGCAGGACCATGTGATCGCG-3′

PCR products were analysed by 1.2% agarose-gel electrophoresis, and purified by Agarose Gel DNA purification kit (TaKaRa, DaLian, China). Purified products were subcloned into pGEM-T vector system (Takara, DaLian, China) following the manufacturer′s instructions. Plasmid DNA was transformed into DH5α-competent cells. Positive clones were sequenced by Invitrogen (Shanghai, China). The fragments from 5′−/3′-RACE were sequenced completely in both directions. The *siltCAT* nucleotide sequence has been submited in GenBank (accession number JQ_663444).

### Sequence Analysis

The coding sequence of *siltCAT* was performed by the EXPASY Proteomics Server (http://expasy.org/) [22]–[24]. Comparative analysis of deduced amino acid sequences with other CATs was performed using protein–protein Blast programs (http://www.ncbi.nlm.nih.gov/BLAST/). The multiple alignment analysis was performed by using the Clustal W software package [25]. Prediction of transit peptide of deduced protein was performed by ChloroP server version1.1 (http://www.cbs.dtu.dk/services/ChloroP/). Prediction of the function of the deduced protein was performed with the conserved domains and protein classification programs (http://www.ncbi.nlm.nih.gov/Structure/cdd/cdd.shtml). The neighbor-joining tree was constructed using the MEGA 5.05 [26] and assessed by the bootstrap test based on 1, 000 replicates.

### Quantitative Real-time PCR and Semi-quantitative RT-PCR

The expression pattern of *siltCAT* gene was further investigated by reverse transcription (RT) PCR and real-time PCR. Total RNA was isolated from various developmental stages of *S. litura* and the collected tissues including fat body, midgut, malpighian tubules and cuticle. All samples were used for reverse transcription to obtain the first-strand cDNA as mentioned above. Real-time PCR was performed in an iCycler iQ Real-Time PCR Detection System (Bio-Rad, USA) with SYBR Premix Ex Taq (Takara, Japan). Each pair of PCR primers were designed to span a cDNA exon-exon border to avoid amplification of potential traces of genomic DNA [27]. The primers of *siltCAT* amplified 108 bp fragments from cDNA (Table 1). As an endogenous control to normalize the results of a variable target gene and to correct for sample-to-sample variation, a β-Actin (DQ___494753) cDNA fragment was amplified with Actin-F and Actin-R primers (Table 1). The products were analysed by sequencing, agarose gel electrophoresis and performing a melting curve, which indicated that each reaction did not produce non-specific amplification. The experimental details about real-time PCR and RT-PCR were conducted according to Liu [28].

Relative quantification was performed by using the comparative 2^−△△CT^ method [29] to investigate the expression pattern of different developmental stages and tissues. All data were normalized to endogenous actin levels from the same individual sample. During the analysis of the relative fold-change, the first-instar larvae sample, which was one of the experimental samples, was taken as the calibrator [30]. Thus, the relative fold-change was assessed by comparing the expression level of *siltCAT* in other developmental stages to that in first-instar. For a valid ΔΔC_T_ calculation with the comparative 2^−△△CT^ method for relative quantification, the amplification efficiencies of the target and reference must be approximately equal. Consequently, the quantity of transcripts was estimated from a standard line derived from 10-fold serial dilutions of cDNA pooled from each sample. In order to test the statistical significance of the observed differences, linear regression analysis was performed using the Probit Regression program in Statistical Package for Social Science (SPSS) Version 17.

### Experiments of siRNA Interference *siltCAT* in SL-1 Cell Line

#### Production of siRNA and transfection

For knockdown of *siltCAT*, the small interfering RNA (siRNA) was designed and synthesized on the basis of the cDNA sequence of the *siltCAT* gene by GeneChem Company (Shanghai, China). The antisense and sense sequences were 5′- GCGGAAAGCUGCAAAUUCATT -3′ and 5′- UGAAUUUGCAGCUUUCCGCTT -3′, respectively. Unrelated siRNA, which is a scramble sequence of the antisense strand with the sequence 5′- AUAUGCGCAACAUUGACA -3′ was synthesized as a negative control. Besides, a positive control (normal cells without siRNA) was also run with the culture plates. siRNA against *siltCAT* and scrambled siRNA were transfected into cells using Turbofect siRNA Transfection Reagent (Fermentas, Lithuania) according to the manufacturer's protocol. All of siRNA were added to the solution to furnish final concentrations of 50 nM and 100 nM, respectively. Each experiment was repeated three times. Subsequently, cells were immediately suspended in complete medium and incubated in a humidified 27°C incubator. After 24–48 h the transcriptional level of *siltCAT* in the above cell line was confirmed by real-time PCR and then cells were used for further studies.

#### Measurement of intracellular ROS

For visualization and analysis of intracellular ROS, the oxidation sensitive probe dihydrochloride acetyl acid dichloride fluorescent yellow (DCFH-DA) (Invitrogen, USA) was used, as previously described [31]. To analyze the net intracellular generation of ROS by using a BD FACS 101 Calibur (Becton and Dickinson Company, USA), cells (2×10^6^) were detached by trypsinization after incubation in the absence or presence of the different factors. The cellular fluorescence intensity was measured after 30 min of incubation with 10 µM DCFH-DA by using flow cytometry. For each analysis, 15,000 events were recorded.

#### Detection of apoptosis

The apoptosis induction effect of RNAi on *S. litura* cultured cell line SL-1 was studied by using various methods according to Huang [32], including DNA fragmentation assay, light microscopy and caspase-3 assay, which provided preliminary studies on the mechanism of apoptosis induction.

#### Cell-cycle analysis

In brief, cells (2×10^6^) at different concentrations and different treatment times were collected and washed in PBS, slowly fixed in 75% ethanol, and kept at −20°C for at least 1 h. The cell pellet was washed again with PBS and centrifuged at 500 g for 5 min. The pellet was resuspended in 200 µl cold PBS and stained in the dark with PI solution (0.1% Triton X-100, 0.1 mM EDTA, 100 µg/ml RNase A, 50 µg/ml PI) for 30 min in ice. The total cellular DNA content was analyzed with a BD FACS 101 Calibur.

### In vivo Assay for the Effect of RNAi-mediated CAT Knockdown on *S. litura*

#### Double-stranded RNA synthesis

For double-stranded RNA (dsRNA) preparation, forward and reverse primers (dsCAT-F and dsCAT-R) containing a T7 promoter site were designed to amplify a 487 bp fragment by PCR using *siltCAT* cDNAs as templates (Table 1). The dsRNA synthesis was carried out using the T7 RiboMAX™ Express RNAi System (Promega, USA) following the manufacturer’s protocol. The amplification reactions protocol comprised 35 cycles of 94°C for 35 s, 58°C for 40 s and 72°C for 45 s, with a final extension step of 72°C for 10 min. The sequence was verified by sequencing (Invitrogen, Shanghai, China). The Green Fluorescent protein (GFP) dsRNA was produced from the GFP gene (ACY56286) and used as a negative injected control. The PCR primers dsGFP-F and dsGFP-R were used to amplify the GFP fragment (688 bp) (Table 1). The dsRNA was purified out of the reaction mixture with a phenol/chloroform extraction followed by an ethanol precipitation. The purity was assessed by 1.2% agarose/ethidium bromide gel electrophoresis and dsRNA was quantified using an Eppendorf BioSpectrometer® kinetic (Eppendorf, Hamburg, Germany). Prior to use dsRNA samples were concentrated to 2.5 µg/µl using an Eppendorf Concentrator plus (Eppendorf, Hamburg, Germany) and stored at −80°C.

#### Double-stranded RNA injections

To deliver dsRNA into the body of *S. litura*, a 175ASN 5 µl Syringe (Hamilton, Bonaduz, Switzerland) was used to inject individual as previously described [33] with some modification. Injections were performed directly into the hemocoel around the abdomen. All individuals were injected with 2 µl of a 2.5 µg/µl stock dsRNA solution (5 µg per individual) for dsCAT (knock down group) and dsGFP (negative control group). Preliminary experiments were performed to determine the optimal dose of dsRNA to be injected (data not shown). For each experiment, 90 *S. litura* larvae were synchronized at the moment of the fourth instar. After injections, these insects were transferred to a growth cabinet with a humidifier at 27°C, using 85% RH and a 16∶8-hlight: dark photoperiod. Non-injected individuals of the same age and kept under the same conditions were used as the positive control.

For the follow-on experiments including RT-qPCR to verify the effectiveness of RNAi and measurement of catalase activity, three pools of fourth instar larvae were collected containing twenty individuals each, from experimental and control groups at every day of the week after injection. RNA extraction and cDNA synthesis were performed as described above.

#### Measurement of catalase activity

Enzyme activity of CAT was spectrophotometrically measured by the method of Aebi [34]. Briefly, samples were collected from fourth-instar larvae and homogenised in 400 µl buffer (50 mM potassium phosphate buffer, pH 7.0, containing 0.1% Triton X-100), centrifuged for 10 min at 2, 500 rpm. The supernatant was transferred to a new tube and kept on ice for remaining experiments. An aliquot (30 µl) of the supernatant was diluted into 470 µl of cold buffer (50 mM potassium phosphate, pH 7.0) and 500 µl of cold 20 mM H_2_O_2_ was added, thus the 1-ml final reaction contained 10 mM H_2_O_2_. After that, the activity of CAT was measured by using the Catalase Assay Kit (Sigma, St. Louis, MO) following the manufacturer’s protocol. Enzyme-specific activities were expressed as units/mg of protein. One unit of CAT activity was defined as 1 µM of H_2_O_2_ consumed per minute at pH 7.0 at 25°C at a substrate concentration of 10 mM H_2_O_2_. The activities were measured spectrophotometrically at 240 nm wavelengths. All assays were carried out in triplicate.

#### Survival assays

Insects were injected with dsRNA of catalase (dsCAT) as described above. Meanwhile, those injected with dsRNA for GFP (dsGFP) and non-injected groups were used as controls. Each experiment for survival analyses was repeated independently three times and three groups of 30 larvae were used for each treatment. To ensure that the observed mortality effects were well within the window of effective RNAi silencing via injection of dsRNA [35], all dead individuals at 24 post-injections were removed and were not included in the experiment. Dead larvae were counted and removed from the cage daily from day 2 to 7 after injection. Mortality was assessed in each cage every day to assess the survival in dsCAT-injected larvae compared to controls.

#### Statistical analysis

Data are expressed as the means ± S.E.M of three independent experiments. SPSS 17.0 software was used to perform *t*-tests to identify significant differences at a 95% confidence level (*p*<0.05). When data followed normal distribution, ANOVA was used to verify difference between groups and a pair-wise *t*-test was used to test differences between the mean of two independent groups. When data distribution was non-normal, Kruskal-Wallis ANOVA was used and the Wilcoxon rank sum was used to test differences between medians of two independent groups. Survival curves were analysed with the Log rank test, as showed in Rogers and Bates [36].

## Results

### Molecular Cloning and Sequence Analysis of *siltCAT*

**Figure 1:**
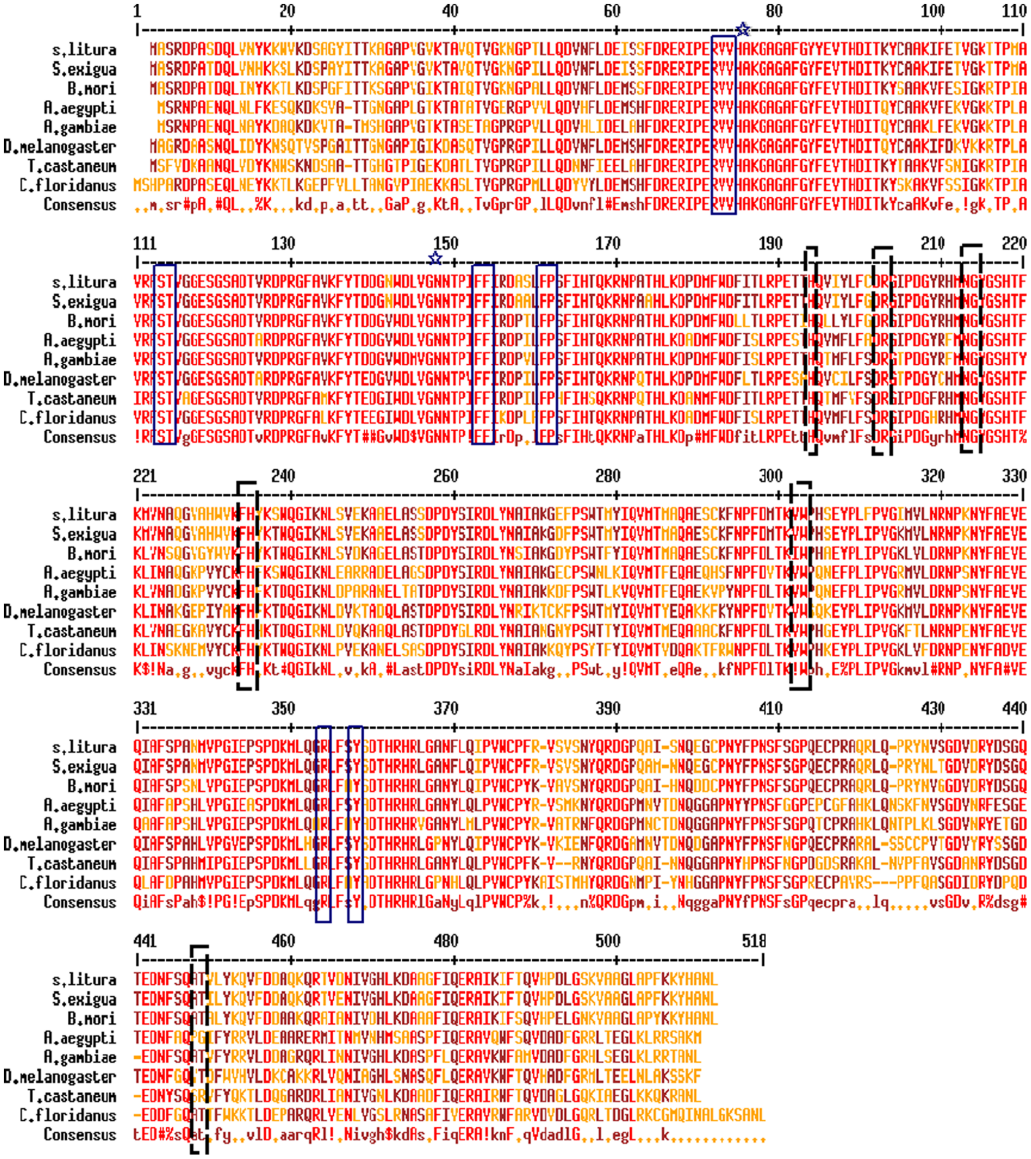
Alignment of amino acid sequences of *siltCAT* and catalase family proteins. Conserved residues in catalases are red, consensus alternatives are brown. The solid blue box and black dotted box mark heme binding and NADPH binding residues, respectively. The symbol **☆** mark residues that define catalytic residues. S. litura *Spodoptera litura* (accession no.: JQ_663444); S. exigua *Spodoptera exigua* (AEP_40969); B. mori *Bombyx mori* (NP_0010369); A. aegypti *Aedes aegypti* (XP_001663600); A. gambiae *Anopheles gambiae* (ABL_09376); D. melanogaster *Drosophila melanogaster* (NP_536731); T. castaneum *Tribolium castaneum* (NP_001153721); C. floridanus *Camponotus floridanus* (EFN_66292).

With degenerate primers based on the consensus sequence of CAT family proteins, a small fragment of conserved intermediate coding region (882 bp) was initially amplified from female adult cDNA of *S. litura* and confirmed by DNA sequencing and the BLAST search. Subsequently, a specific primer was designed based on this sequence and used in combination with primer supplied with the kit to obtain the fragment of the N-terminal (423 bp) and C-terminal (784 bp) coding region, respectively. The full-length cDNA clone (named *siltCAT*) encodes a protein of 507 amino acids in length with molecular mass of 56.87 kDa and isoelectric point of 8.06, without a signal peptide and mitochondrial or peroxisomal targeting sequences of types 1 and 2. *siltCAT* sequence contains the conserved residues His75 and Asn148 (catalytic), Val73, Val74, Ser112, Thr113, Phe151, Phe152, Phe169, Pro170, Arg352 and Tyr356 (putative heme binding sites) and His192, Asp200, Arg201, Asn211, Gly212, Phe232, His233, Val300, Trp301, Ala443 and Thr444 (putative NADPH binding sites) (Fig. 1). BLAST analysis revealed that *siltCAT* showed 68–96% amino acid identity with CAT family proteins of various insect species across different orders. Specifically, *siltCAT* displays the highest degree of identity to *Spodoptera exigua* (96%) and relatively lower identities with *Bombyx mori* (87%), *Drosophila melanogaster* (72%), and *Tribolium castaneum* (70%). Phylogenetic analysis of *siltCAT* using a neighbor-joining tree indicated the *S. litura* forms a clade with *S. exigua*, suggesting that these CAT proteins share the highest sequence similarity (Fig. S1).

### Developmental and Tissue Specific Expression of *siltCAT*

**Figure 2:**
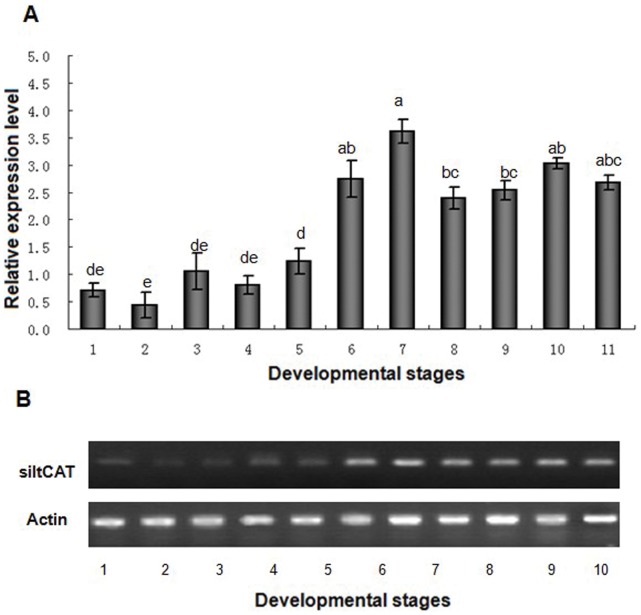
*SiltCAT* transcript levels in different developmental stages were determined by RT-qPCR (A) and RT-PCR (B). The expression of *SiltCAT* in the first-instar larvae sample was taken as the calibrator and the mRNA level was normalized relative to the β-Actin transcript. The standard error of the mean for three technical replicates is represented by the error bar. Different letters above bars indicate significant differences between different developmental stages (*P*<0.05). 1, Egg; 2, Newly hatched larva; 3, first-instar larva; 4, second-instar larva; 5, third-instar larva; 6, forth-instar larva; 7, fifth-instar larva; 8, Prepupa; 9, Pupa; 10, Female adulte; 11, male adulte.

**Figure 3:**
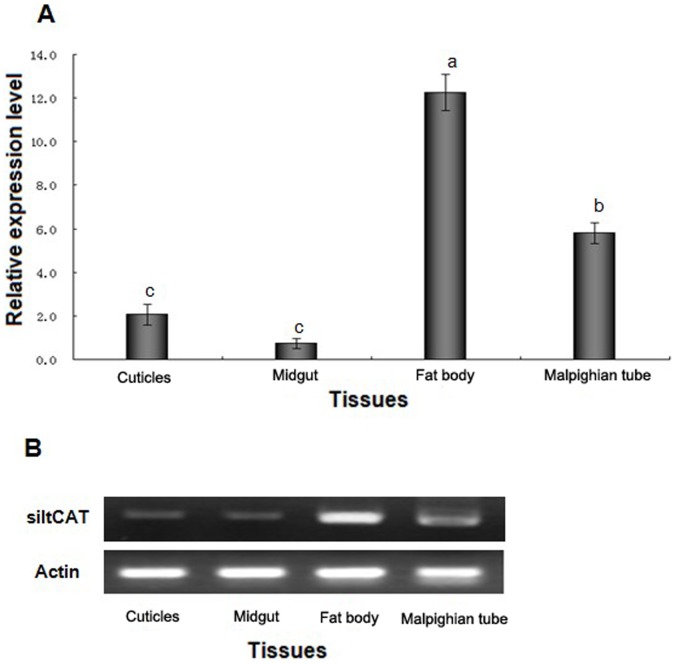
*SiltCAT* transcript levels in various tissues of fifth-instar larvae were determined by RT-qPCR (A) and RT-PCR (B). The expression of *SiltCAT* in the midgut sample was taken as the calibrator and the mRNA level was normalized relative to the β-Actin transcript. The standard error of the mean for three technical replicates is represented by the error bar. Different letters above bars indicate significant differences between different tissues (*P*<0.05).

Real-time PCR and Semi-quantitative RT-PCR were used to characterize the expression of *siltCAT* gene at all developmental stages and various tissues of the fifth-instar larvae. All samples were normalized to β-Actin. The absolute values of the slope of all lines from template dilution plots (log cDNA dilution versus △C_T_) were close to zero. Therefore, the amplification efficiencies of the target and reference genes were similar in our analysis. The results demonstrated that *siltCAT* mRNA (Fig. 2A & Fig. 2B) was highly expressed in the fourth-instar larva, fifth-instar larva, pre-pupa and adult, but little expression was obtained in egg, newly hatched larva, first-instar larva, second-instar larva and third-instar larva. Simultaneously, *siltCAT* mRNA was detected in all the tested tissues, but the levels of expression showed significant difference. The highest expression of *siltCAT* in the fat body was nearly 16 times more than the lowest expression in the midgut (Fig. 3A & Fig. 3B).

### Consequences of *siltCAT* RNAi Knockdown in SL-1 Cells

#### Down-regulation of *siltCAT* mRNA by RNAi

**Figure 4:**
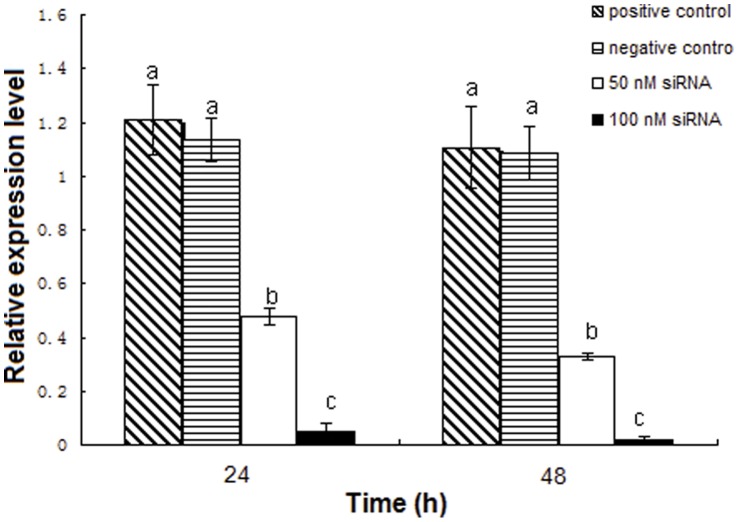
The relative mRNA expression levels of *siltCAT* gene after RNAi in SL-1 cells. Effect of different doses of siRNA (final concentrations of 50 nM and 100 nM) on the expression levels of *siltCAT* was detected by RT-qPCR after 24 h and 48 h treatment. Positive control, normal cells without siRNA; negative control, cells treated with unrelated siRNA. The data represent the mean values ± S.E.M of three independent experiments. Different letters above bars indicate significant differences between different treatments at the same time (*P*<0.05).

To investigate the efficiency of RNAi after transfection of siRNA in SL-1 cells, *siltCAT* mRNA relative levels were measured by qRT-PCR. Two different doses of siRNA were used to evaluate the knockout effect of *siltCAT* in SL-1 cells. Compared with the blank control group, the transcript levels of *siltCAT* decreased by 42.28%–64.83% after transfection with final concentration of 50 nM siRNA and decreased by 94.34%–98.57% after transfection with final concentration of 100 nM siRNA, respectively (Fig. 4). The result showed that *siltCAT* was efficiently silenced by siRNA in SL-1 cells.

#### Accumulation of SL-1 intracellular ROS by RNAi in vitro

**Figure 5:**
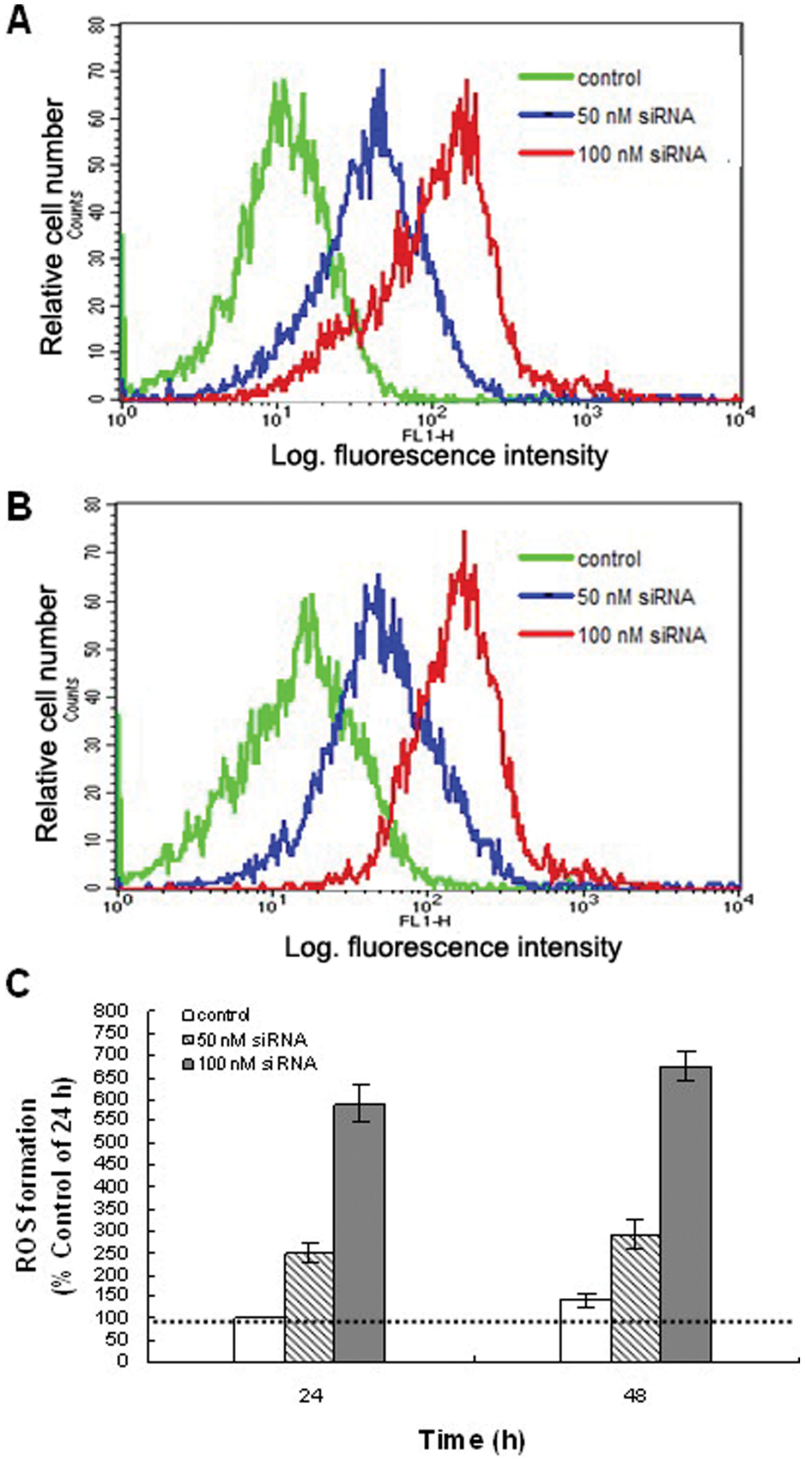
Change of intracellular ROS post transfection in vitro. The levels of ROS were determined using the dye DCFH-DA. Flow cytometric histograms showed a broad unimodal distribution of DCF fluorescence in different cell groups. Increased fluorescence in the FL1-H channel indicates increased levels of ROS. The control is representative of different controls including positive and negative control groups. (A) Increased intracellular ROS in SL-1 cells after 24 h treatment. (B) Increased intracellular ROS in SL-1 cells after 48 h treatment. As shown in (A) and (B), ROS increased significantly in all cell groups post transfection. The levels of ROS are higher in the cells transfected with *siltCAT* siRNA, *P*<0.05 vs Control group. (C) The change of intracellular ROS levels at different observation points. The ROS levels are higher in the cells transfected with *siltCAT* siRNA at the two observation points (*P*<0.05 vs Control group), particularly in the cells transfected with 100 nM siRNA (*P*<0.05 vs 50 nM siRNA groups).

Evidence that apoptosis can be induced by oxidative stress is provided by studies in which mediators of apoptosis can either be induced by ROS or inhibited by the addition of antioxidants [37]. Flow cytometer was used to examine ROS production and apoptosis after *siltCAT* gene knockdown. The intracellular ROS level was measured with DCFH-DA as a fluorescent probe of ROS, which can produce an enhanced fluorescence when cells generate ROS. No significant difference in ROS levels was observed between positive control groups and negative control groups (data not shown). However, compared with the negative control (treated with unrelated siRNA), the fluorescence of intracellular ROS production increased from 148.40% to 489.74% with increasing dosage for 24 h. As well, the fluorescence increased from 190.60% to 575.11% with increasing dosage for 48 h (Fig. 5A & 5B). These results showed that intracellular ROS increased significantly in all cell groups after treated with siRNA, especially in 100 nM siRNA group. Fig. 5C showed that, in the same operation time, ROS production in SL-1 cells treated with siRNA of different concentrations increased markedly. On the contrary, intracellular ROS production did not increase significantly with the time pronging at certain concentration. The ROS levels increased rapidly and reached the point at 24 h, while tended to slow at 48 h under the same concentration.

#### *siltCAT* knockdown induced cell apoptosis and cycle arrest in SL-1 cells

**Figure 6:**
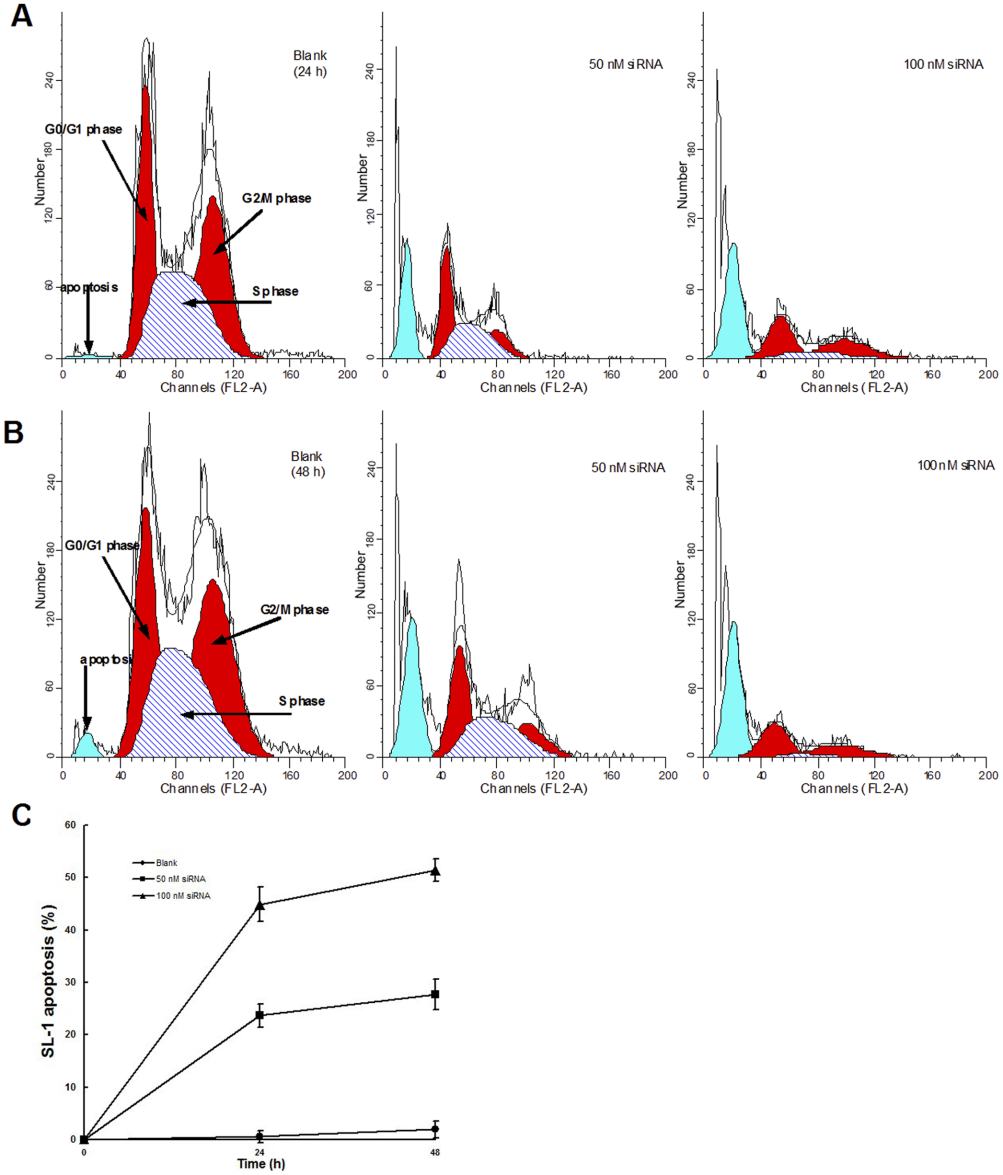
Effect of *siltCAT* knockdown on apoptosis and cell cycle distribution in SL-1 cells. The apoptosis rates were determined using the dye PI. Flow cytometric profiles showed changes of cell cycle and apoptosis in different cell groups. (A) It is shown that knockdown of *siltCAT* with 50 nM siRNA and 100 nM siRNA changed cell cycle and increased apoptosis after 24 h, respectively. The graph is representative of three independent experiments. (B) It is shown that knockdown of *siltCAT* with 50 nM siRNA and 100 nM siRNA changed cell cycle and increased apoptosis after 48 h, respectively. Representative images are shown. (C) The change of SL-1 apoptosis at different observation points. Data are means ± S.E.M of three independent experiments. The apoptosis rate are higher in the cells transfected with 50 nM siRNA or 100 nM siRNA at the two observation points (*P*<0.05 vs Blank group), particularly in the cells transfected with 100 nM siRNA (*P*<0.05 vs Blank and 50 nM siRNA groups).

Fig. S2 showed DNA Ladder formation or DNA fragmentation after RNAi, DNA Ladder was observed clearly from 24 to 48 h after treatments, by using agarose gel electrophoresis. Controls did not exhibit any DNA Ladder that may be no apoptosis or low sensitivity of electrophoresis. In addition, apoptotic bodies indicated typical apoptosis morphological changes (Fig. S3) as well as significant activation of caspase-3 occurred during apoptosis (Fig. S4). To further analyze whether the SL-1 apoptosis resulted from impaired cell proliferation, the cell-cycle profile was examined by nuclear DNA staining with Propidium iodide (PI) using flow cytometry (Fig. 6A & 6B). The cell cycle distributions were determined at the indicated siRNA concentrations. Table S1 shows 24 h and 48 h after treated at a concentration of 50 nM, knockdown of *siltCAT* resulted in significant increase in the proportion of cells in the G0/G1 phase with concomitant decreases in the S phase and G2/M phase when compared with control. However, when *siltCAT* gene were almost completely silenced by a concentration of 100 nM siRNA at 24 h and 48 h, the proportion of cells in both G0/G1 phase and G2/M phase increased significantly with sharp decrease in the S phase (Table S1). Furthermore, from 24 to 48 h, there was not obvious change either in cell cycle phase or apoptosis ratio (Table S1 & Fig. 6C). With the increase of the siRNA concentration, knockdown of *siltCAT* at 24 h and 48 h caused significant enhancement in apoptosis (8.68-, 11.72- to 22.35-, 26.27-fold) when compared with control, respectively (Fig. 6C). Together, these results indicate that cells were arrested at the G0/G1 and G2/M phases to repair RNAi-induced DNA damage and that some of the damage was not repaired causing the cells to undergo apoptosis.

### The Effect of dsCAT-injection on *S. litura*

#### Quantitative analyses of the mRNA and enzyme activity after injection-based RNAi

**Figure 7:**
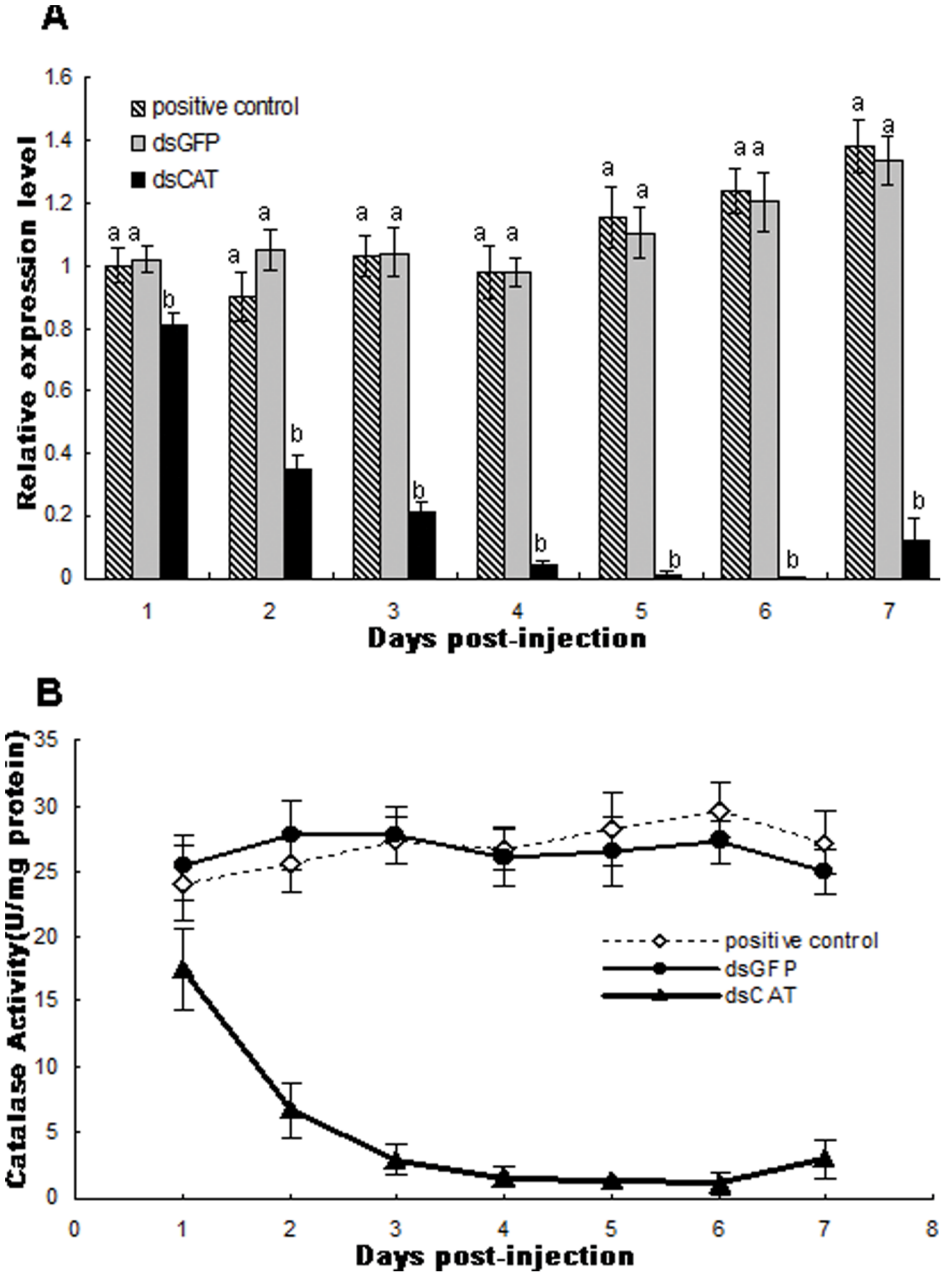
Change in catalase during the experiments after dsRNA-injections. (A) *siltCAT* relative expression levels for dsCAT-injected and dsGFP-injected insects measured by RT-qPCR from day 1 until day 7 after dsRNA injection. The expression of *SiltCAT* in the forth-instar larvae sample was taken as the calibrator. The data represent the mean values ± S.E.M of three independent experiments. Different letters above bars indicate significant differences between different treatments at the same time (*P*<0.05). (B) Catalase activity in dsRNA-injected fourth-instar larvae of *S. litura.* Insects injected with *dsGFP*, *dsCAT* and non-injections were assayed for catalase activity daily for seven days. dsCAT-injected insects had significantly lower activity compared to the dsGFP and uninjected controls after two days (*P*<0.05, *T*-test). Positive control, Non-injected insects; dsGFP, 2.5 µg/µl *dsGFP* treated as a negative control; dsCAT, 2.5 µg/µl *dsCAT* treated.

To better obtain a quantitative measure of the efficiency of injection for RNAi, we detected the transcript level of *siltCAT* using qRT-PCR and measured the level of enzyme activity using a colorimetric method. Fourth-instar larvae that were used in the qPCR analysis were continuously collected 1–7 days after injection. qPCR analysis revealed that the mRNA abundance of *siltCAT* decreased by only 25.34% at the first day whereas the reduction varied from 74.58% to 98.74% two days later, compared with the control (dsGFP-injection groups) (Fig. 7A). However, on the 7th day, the mRNA abundance of *siltCAT* had a slight increase that could be due to the ability of organism to repair itself [38]. The level of CAT enzyme activity also declined significantly from the 2nd day to 7th day after injection, as compared to the negative and positive control groups (Fig. 7B). However, between the fifth day and the sixth day after dsCAT-injection there was still low amount of CAT activity in CAT-silenced individuals. It was probably due to the few transcripts that RNAi failed to degrade and/or to low amounts of protein present before injection because RNAi can interfere in protein translation but not destroy mature proteins. This result confirmed that the CAT gene was susceptible to silencing by RNAi using the injection method.

#### Knockdown of *siltCAT* resulted in mortality of *S. litura*

**Figure 8:**
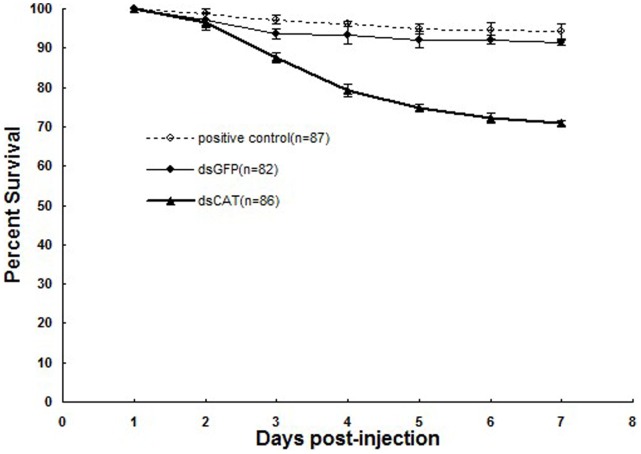
Mean survival of *S. litura* at different days post-injection. The mortality of animals injected with *dsGFP*, *dsCAT* and un-injections groups was monitored daily from day 1 up to day 7 after injection. The data represent the mean values ± S.E.M of three independent experiments. Different letters above bars indicate significant differences between different treatments at the same time (*P*<0.05). Positive control, Non-injected insects; dsGFP, 2.5 µg/µl *dsGFP* treated as a negative control; dsCAT, 2.5 µg/µl *dsCAT* treated.

In order to investigate if CAT intervention was implicated in developmental mortality, *siltCAT* was depleted via RNAi injection in fourth-instar larvae of *S. litura* and mortality was recorded from day 1 up to day 7 after injections. In the survival curves (Fig. 8), approximately starting from the third day after dsRNA-injection, the percentage of surviving dsCAT-injected larvae gradually declined. Mortality rate was higher in knocked down (dsCAT) *S. litura*, compared to that of dsGFP-injected and non-injected groups. Therefore, the results indicated that CAT expression has a significant role in *S. litura* survival during the development.

## Discussion

CAT has long been postulated to represent a main line of defense against oxygen free radical induced damage by scavenging H_2_O_2_ and thereby limiting the formation of the highly reactive hydroxyl radical. However, biochemical information on CAT of insect species is still insufficient. In this paper, cDNA sequence was first cloned and characterized from the Lepidoptera insect *S. litura* encoding *siltCAT*. The calculated molecular size of *siltCAT* is 56.87 kDa. This size was similar to those of other CATs isolated so far [20]. Fig. 1 showed that deduced amino acid sequence shared high homology with CATs of other insects, especially the conserved residues that defined heme and NADPH orientation. Heme-containing CATs have been identified in organisms from bacteria to humans [39], [40]. The catalytic mechanism is a reaction of two-step. In the first step, the heme Fe^3+^ reduces a hydrogen peroxide molecule to water and generates a covalent Fe^4+^ = O oxyferryl species with a porphyrin p-cation radical [41], referred to as compound I. In the second step, compound I oxidizes a second peroxide molecule to molecular oxygen and releases the ferryl oxygen species as water. NADPH is tightly bound to several CATs [42]. However, the detailed mechanism of NADPH action is unclear. Recent experiments suggested that twelve amino acid residues were responsible for the binding of NADPH in the human erythrocyte CAT [43]. Nine of the binding residues were also identified in the sequence of *siltCAT* (Fig. 1). Our successful cloning and analyzing of *siltCAT* provided a basis for further characterization of CAT properties and biological functions in *S. litura.*

To better understand the expression pattern of CAT, qPCR and RT-PCR were used to determine the expression level of the isolated *siltCAT* and its role in various developmental stages and tissues. Pattern analysis suggested that the expression level of *siltCAT* mRNA in *S. litura* was distributed mainly over last instar larvae, pupa and adult (Fig. 2A & 2B), this maybe because it regulated different physiological processes in specific development phases. Considering the function of CAT as a scavenger of ROS, the ubiquitous distribution of *siltCAT* was reasonable. The excessive ROS generated from the rapidly increased metabolism during the maturation of the larvae dictates the demand of an elevated antioxidant system. Besides, it was during the larval stage on accumulation of substances almost occupying the whole cytoplasm of the cell. During the immature phase metabolic mobilization began in fat body, and during the metamorphosis to adult, the reserves were intensely mobilized [44]. Thus a high level of *siltCAT* expression in the fat body was detected, which may function to balance the oxidative stress. Moreover, there was *siltCAT* expression in midgut, cuticle and the malpighian tubes (Fig. 3A & 3B). Deeper investigations are needed for better understanding of the link between CAT expression pattern and the metabolism needs.

ROS are usually a side product of general metabolism. In the present study, we found that *siltCAT* gene knock-down through RNAi led to significant elevation of intracellular ROS level and apoptosis rate. Furthermore, the more *siltCAT* were silenced, the more intracellular ROS remained unscavenged (Fig. 5). Thus, the knockdown of *siltCAT* level in SL-1 cells contributes to the increased intracellular ROS. The significance of ROS in intracellular signaling has been well documented [45]. Diverse stimuli that increase intracellular oxygen radicals can evoke many cellular events, such as gene activation, cell cycle arrest and apoptosis. Actually, apoptosis and proliferation were intimately coupled. Some cell cycle regulators could influence both cell division and programmed cell death [46]. One of the focus in this study is the relationship between apoptosis induction and cell cycle arrest by knockdown of CAT gene, which was not elaborated previously. In CAT gene knock-down cells, CAT expression was associated with cell cycle phase distribution, meanwhile the rapid decrease of CAT activity led to cell apoptosis in response to H_2_O_2_. Treated cells showed typical apoptosis morphological changes including DNA fragmentation and apoptotic bodies as well as significant activation of caspase-3 (Fig. S2, Fig. S3 & Fig. S4). These results observed clearly support that apoptosis of SL-1 cells was induced by knockdown of CAT gene. The maximum enhancement in apoptosis of each concentration was 11.72-fold and 26.27-fold than control, respectively (Fig. 6C). Within the concentrations of 50–100 nM, RNAi seemed to induce apoptotic cell death in a dose-dependent manner. This result was also consistent with cell cycle analysis by flow cytometry (Fig. 6A & Fig. 6B). After treated at a concentration of 50 nM siRNA, cell cycle phases were redistributed in *siltCAT* knock-down cells, that is, G1 checkpoint arrested, more cells blocked in G0/G1 phase with concomitant decreases both in the S phase and G2/M phase (Table S1). The result confirmed that the arrest of cells in the G0/G1 phase of the cell cycle was an important component of the cellular response to stress. Similarly, reactive nitrogen species (RNS) act as physiologic second messengers like ROS in many cell signaling processes, including immune responses, cell cycle progression and apoptosis. It was reported that RNS could block cell cycle re-entry through sustained production of H_2_O_2_, resulting in cell cycle arrest at the boundary between G0 and G1. Further more, loading cells with CAT not only prevented the accumulation of intracellular H_2_O_2_, but also rescued cell cycle progression from inhibition by RNS through recovering S phase entry [47]. In addition, as temperature-shift experiments in growing rat fibroblasts expressing the A135 V mutant showed that p53 blocked cellular proliferation primarily by inducing arrest in G0/G1, whereas very few cells were arrested in S phase and G2/M [48]. Many studies have also found that proliferation of various cell types could be inhibited through inducing a G0/G1 cell cycle arrest [49], [50]. These phenomena have been described earlier as the development of an aberrant mitotic exit into ‘multinucleate state’, which eventually progresses into apoptosis. There has been no report so far on the CAT-mediated ROS formation causing G0/G1 phase arrest as a key apoptotic mechanism by RNAi in SL-1 cells, and the findings from our study offered further insights into the molecular mechanism underlying CAT knockdown-induced apoptosis. Besides, when the concentration of siRNA increased to 100 nM, in other words, *siltCAT* gene were almost completely silenced (Fig. 4), the proportion of cells both in the G0/G1 phase and G2/M phase increased significantly while the proportion decreased sharply in the S phase (Table S1). As it is well documented that generation of intracellular ROS may lead to DNA damage [51], the observation that cells arrested simultaneously at the G0/G1 and G2/M phases may be because of that more RNAi-induced DNA damage was in need of repair. The early response to DNA damage is to arrest cell cycle progression to allow time for repair of the damage [52]. However, when cells are unable to repair their damaged DNA, they may undergo apoptosis. Knockdown of *siltCAT* in SL-1 cells resulted in a significant increase in the proportion of cells in the G0/G1 and G2/M phases with a concomitant decrease in S phase followed by an increase in apoptosis. By comparison, when *siltCAT* gene was not completely silenced (Fig. 4) there was still some *siltCAT* responsible to clear intracellular ROS, less DNA damage triggered only the G0/G1 phase arrest followed by a lower ratio in apoptosis. Likewise, citrinin-generated ROS in mouse skin that caused cell cycle arrest at the G2/M as well as G0/G1 phases and caused apoptosis because some of the damage was not repaired [53].

Generation of ROS represents one of the accompanying phenomena during oxygen metabolism in aerobes. Intracellular ROS can lead to the alterations in the structure of constitutive cellular molecules through a series of chain radical reactions. These structural alterations are termed oxidative injuries [54]. As mentioned before, regulative mechanism of ROS varies significantly in mammals and insects. ROS produced in tissues of mammals is regulated by their elimination through coordinate action and enzymatic (SOD, CAT, GPX, GSTs-glutathione-S-transferases and GR-glutathione reductase) and non-enzymatic (glutahione, ascorbate and tocopherols) antioxidant components. However, insect species lack selenium-dependent GPX and instead active enzymatic ascorbate recycling pathway serves to eliminate hydrogen peroxide [55], [56]. Due to its higher affinity for H_2_O_2_ compared to CAT, GPX is considered to be very important in the maintenance of the cellular homeostasis in mammalian tissues. Since insects do not contain such functionally important component, the organization of protection against oxidative injuries in insect species remains to be elucidated. Furthermore, unlike mammals, the sites of ROS generation in insect cells are compartmentalized and changed within the cells. Subcellular distribution of antioxidant components in insects clearly differs from that occurring in mammals. The most striking difference is related to CAT which is widely distributed at subcellular level in insects, while in mammalian species this enzyme is dominantly localized in peroxisomes [57]. Such a wide subcellular distribution of CAT in insect species could be explained by the absence of selenium-dependent GPX. Thereby CAT plays an extensive and paramount role in ROS regulation in insects. Indeed, evidence mounted that inactivation or silencing of CAT in *Musca domestica* [58], *Rhodnius prolixus* [9], *Drosophila melanogaster* [8], *Anopheles gambiae* [17] and *Lutzomyia longipalpis* [24] led to increased mortality. In our results, the silencing of the CAT antioxidant enzymes in *S. litura* led to increased mortality (Fig. 8), which further highlighted the indispensable role of CAT in the development of insect. It is likely that the impaired ROS processing capacity in insects due to inactivation or silencing of CAT leads to increased susceptibility of these insects to H_2_O_2_ challenge and to higher levels of endogenous molecular H_2_O_2_.

In addition, the application of RNA interference to control pests has been explored by many researchers [59]–[61], but it is difficult to select the suitable insect specific target genes, or transcription factors, in order to achieve successful pest management [15]. Although many insect genes have essential functions, it is unlikely that random gene knockdown or silencing by siRNAs will be effective in killing insects [62], [63]. This study shows that CAT is a key gene in determining the survival of *S. litura*. Future developments and improvement are anticipated to explore the intervention of this gene as a potential target for field-level control of *S. litura*.

## Supporting Information

Figure S1 **Neighbor-joining tree of** ***siltCAT*** **and sequences showing a high degree of identity to this protein.** S. litura, *Spodoptera littoralis* (accession no.: AFG_31725.1); S. Exigua, *Spodoptera exigua* (AEP_40969); B. mori, *Bombyx mori* (NP_0010369); D. melanogaster, *Drosophila melanogaster* (NP_536731); C. idella, *Ctenopharyngodon idella* (ACL_99859.2); P. perniciosus, *Phlebotomus perniciosus* (ADH_94605.1); L. longipalpis, *Lutzomyia longipalpis* (ABV_60342.1); C. quinquefasciatus, *Culex quinquefasciatus* (XP_001848573.1); P. vanderplanki, *Polypedilum vanderplanki* (ADM_64337.1); T. castaneum *Tribolium castaneum* (NP_001153721); B. plicatilis, *Brachionus plicatili s*(BAG_28837.1); G. morsitans, *Glossina morsitans morsitans* (ADD_20421.1); C. plicata, *Cristaria plicata* (ADM_64337.1); C. farreri, *Chlamys farreri* (ABI_64115.1); P. fucata, *Pinctada fucata* (ADW_08700.1); A. aegypti, *Aedes aegypti* (XP_001663600); A. gambiae *Anopheles gambiae* (ABL_09376); F. chinensis, *Fenneropenaeus chinensis* (ABW_82155.1); L. vannamei, *Litopenaeus vannamei* (AAR_9998.1); C. floridanus, *Camponotus floridanus* (EFN_66292); H. saltator, *Harpegnathos saltator* (EFN_78714.1); D. rerio, *Danio rerio* (AAF_89686.1); M. undulatus, *Melopsittacus undulatus* (AAO_72713.1); M. musculus, *Mus musculus* (BAC_36005.1); H. sapiens, *Homo sapiens* (NP_001743.1). The phylogenetic tree was constructed using MEGA 5.05 presented with 70% cut-off bootstrap value.
(DOC)

Figure S2 **Agarose gel electrophoresis analysis of genomic DNA fragmentation of SL-1 cells treated by siRNA.** 1: At 48 h after cells untreated with siRNA as a positive control, showed no DNA fragmentation; 2: At 48 h after treated with unrelated siRNA as a negative control, showed no DNA fragmentation; 3–4: At 24 h after treatment with 50 nM and 100 nM siRNA, respectively, showed DNA fragmentation; 5–6: At 48 h after treatment with 50 nM and 100 nM siRNA, respectively, showed DNA fragmentation.
(DOC)

Figure S3 **Morphological study by inverted phase contract microscope (200×).** Parts (a) and (d) were cells treated with unrelated siRNA for 24 and 48 h, respectively. Parts (b) and (e) were cells treated with 50 nM siRNA for 24 and 48 h, respectively. Parts (c) and (f) were cells treated with 100 nM siRNA for 24 and 48 h, respectively.
(DOC)

Figure S4 **Increase of caspase-3 activity in SL-1 cells after treated with siRNA at different time points.** Positive control: untreated with siRNA. Negative control: treated with unrelated siRNA. Data are expressed as arithmetic means ± S.E.M of three independent experiments. Treatment means sharing the same letter are not significantly different from each other (Tukey’s tests, *P*<0.05).
(DOC)

Table S1 **Different cell cycle phases of SL-1 cells treated with siRNA.**
(DOC)
