# Supplementary material for: RNAi-Mediated Knockdown of Catalase Causes Cell Cycle Arrest in SL-1 Cells and Results in Low Survival Rate of Spodoptera litura (Fabricius)
Source: PLoS One. 2013 Mar 26;8(3):e59527. doi: 10.1371/journal.pone.0059527 (PMC3608696; doi:10.1371/journal.pone.0059527)
Supplement: Figure S1 — Neighbor-joining tree of siltCAT and sequences showing a high degree of identity to this protein. S. litura, Spodoptera littoralis (accession no.: AFG_31725.1); S. Exigua, Spodoptera exigua (AEP_40969); B. mori, Bombyx mori (NP_0010369); D. melanogaster, Drosophila melanogaster (NP_536731); C. idella, Ctenopharyngodon idella (ACL_99859.2); P. perniciosus, Phlebotomus perniciosus (ADH_94605.1); L. longipalpis, Lutzomyia longipalpis (ABV_60342.1); C. quinquefasciatus, Culex quinquefasciatus (XP_001848573.1); P. vanderplanki, Polypedilum vanderplanki (ADM_64337.1); T. castaneum Tribolium castaneum (NP_001153721); B. plicatilis, Brachionus plicatili s(BAG_28837.1); G. morsitans, Glossina morsitans morsitans (ADD_20421.1); C. plicata, Cristaria plicata (ADM_64337.1); C. farreri, Chlamys farreri (ABI_64115.1); P. fucata, Pinctada fucata (ADW_08700.1); A. aegypti, Aedes aegypti (XP_001663600); A. gambiae Anopheles gambiae (ABL_09376); F. chinensis, Fenneropenaeus chinensis (ABW_82155.1); L. vannamei, Litopenaeus vannamei (AAR_9998.1); C. floridanus, Camponotus floridanus (EFN_66292); H. saltator, Harpegnathos saltator (EFN_78714.1); D. rerio, Danio rerio (AAF_89686.1); M. undulatus, Melopsittacus undulatus (AAO_72713.1); M. musculus, Mus musculus (BAC_36005.1); H. sapiens, Homo sapiens (NP_001743.1). The phylogenetic tree was constructed using MEGA 5.05 presented with 70% cut-off bootstrap value. (DOC) [file pone.0059527.s001.doc]

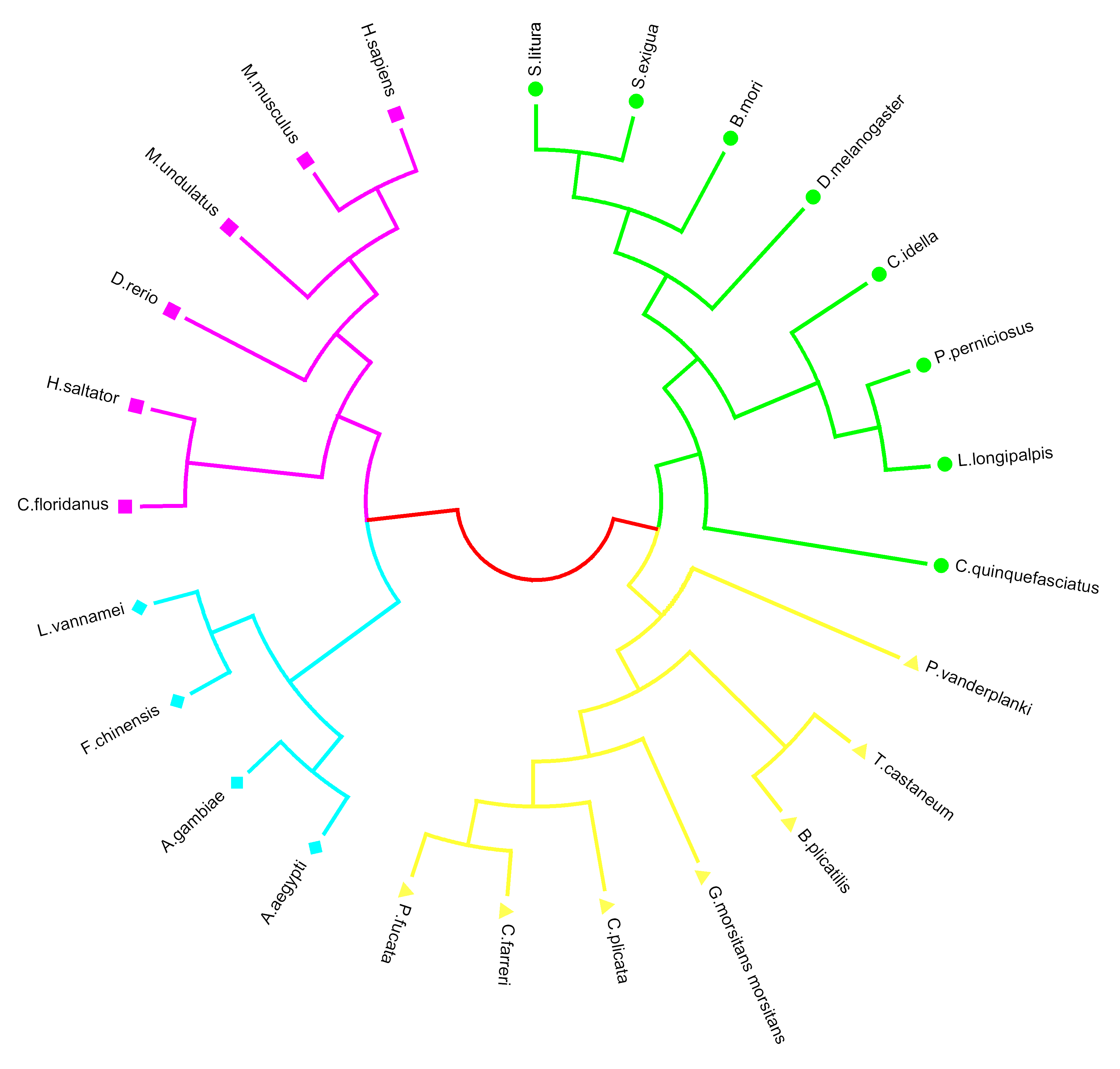


**Figure S1 Neighbor-joining tree of *siltCAT* and sequences showing a high degree of identity to this protein.** S. litura, *Spodoptera littoralis* (accession no.: AFG­_31725.1); S. Exigua, [*Spodoptera exigua*](app:ds:Spodoptera exigua) (AEP_40969); B. mori, [*Bombyx mori*](app:ds:Bombyx mori mori) (NP_0010369); D. melanogaster, *Drosophila melanogaster* (NP_536731); C. idella, *Ctenopharyngodon idella* (ACL_99859.2); P. perniciosus, *Phlebotomus perniciosus* (ADH_94605.1); L. longipalpis, *Lutzomyia longipalpis* (ABV_60342.1); C. quinquefasciatus, *Culex quinquefasciatus* (XP_001848573.1); P. vanderplanki, *Polypedilum vanderplanki* (ADM_64337.1); [T. castaneum](app:ds:Tribolium castaneum) [*Tribolium castaneum*](app:ds:Tribolium castaneum) (NP_001153721); B. plicatilis, *Brachionus plicatili s*(BAG_28837.1); G. morsitans, *Glossina morsitans morsitans* (ADD_20421.1); C. plicata, *Cristaria plicata* (ADM_64337.1); C. farreri, *Chlamys farreri* (ABI_64115.1); P. fucata, *Pinctada fucata* (ADW_08700.1); A. [aegypti](app:ds:aedes aegypti), [*Aedes aegypti*](app:ds:aedes aegypti) (XP_001663600); [A. gambiae](app:ds:Anopheles gambiae) [*Anopheles gambiae*](app:ds:Anopheles gambiae) (ABL_09376); F. chinensis, *Fenneropenaeus chinensis* (ABW_82155.1); L. vannamei, *Litopenaeus vannamei* (AAR_9998.1); [C. floridanus](app:ds:Camponotus floridanus), [*Camponotus floridanus*](app:ds:Camponotus floridanus) (EFN_66292); H. saltator, *Harpegnathos saltator* (EFN_78714.1); D. rerio, *Danio rerio* (AAF_89686.1); M. undulatus, *Melopsittacus undulatus* (AAO_72713.1); M. musculus, *Mus musculus* (BAC_36005.1); H. sapiens, *Homo sapiens* (NP_001743.1). The phylogenetic tree was constructed using MEGA 5.05 presented with 70% cut-off bootstrap value.
