# Supplementary material for: RNAi-Mediated Knockdown of Catalase Causes Cell Cycle Arrest in SL-1 Cells and Results in Low Survival Rate of Spodoptera litura (Fabricius)
Source: PLoS One. 2013 Mar 26;8(3):e59527. doi: 10.1371/journal.pone.0059527 (PMC3608696; doi:10.1371/journal.pone.0059527)
Supplement: Figure S2 — Agarose gel electrophoresis analysis of genomic DNA fragmentation of SL-1 cells treated by siRNA. 1: At 48 h after cells untreated with siRNA as a positive control, showed no DNA fragmentation; 2: At 48 h after treated with unrelated siRNA as a negative control, showed no DNA fragmentation; 3–4: At 24 h after treatment with 50 nM and 100 nM siRNA, respectively, showed DNA fragmentation; 5–6: At 48 h after treatment with 50 nM and 100 nM siRNA, respectively, showed DNA fragmentation. (DOC) [file pone.0059527.s002.doc]

**
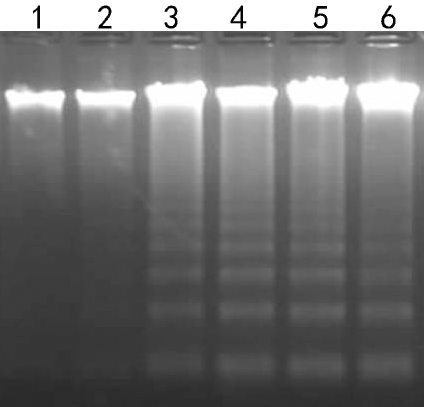
**

**Figure S2 Agarose gel electrophoresis analysis of genomic DNA fragmentation of SL-1 cells treated by siRNA.**

1: At 48h after cells untreated with siRNA as a positive control, showed no DNA fragmentation; 2: At 48h after treated with unrelated siRNA as a negative control, showed no DNA fragmentation; 3-4: At 24 h after treatment with 50 nM and 100 nM siRNA, respectively, showed DNA fragmentation; 5-6: At 48 h after treatment with 50 nM and 100 nM siRNA, respectively, showed DNA fragmentation.
