# Supplementary material for: RNAi-Mediated Knockdown of Catalase Causes Cell Cycle Arrest in SL-1 Cells and Results in Low Survival Rate of Spodoptera litura (Fabricius)
Source: PLoS One. 2013 Mar 26;8(3):e59527. doi: 10.1371/journal.pone.0059527 (PMC3608696; doi:10.1371/journal.pone.0059527)
Supplement: Figure S3 — Morphological study by inverted phase contract microscope (200×). Parts (a) and (d) were cells treated with unrelated siRNA for 24 and 48 h, respectively. Parts (b) and (e) were cells treated with 50 nM siRNA for 24 and 48 h, respectively. Parts (c) and (f) were cells treated with 100 nM siRNA for 24 and 48 h, respectively. (DOC) [file pone.0059527.s003.doc]

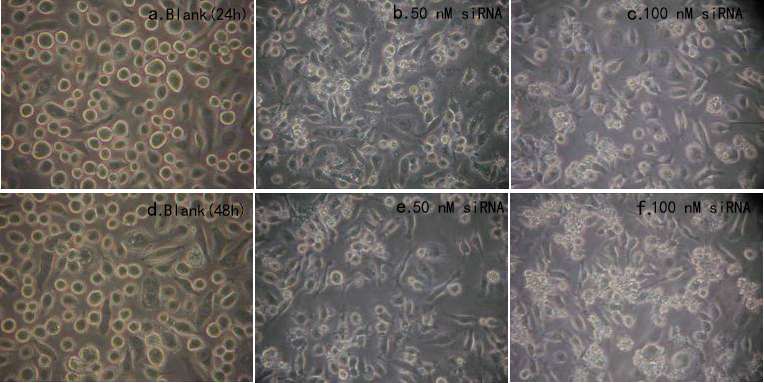


**Figure S3 Morphological study by inverted phase contract microscope (200×).**

Parts (a) and (d) were cells treated with unrelated siRNA for 24 and 48 h, respectively. Parts (b) and (e) were cells treated with 50 nM siRNA for 24 and 48 h, respectively. Parts (c) and (f) were cells treated with 100 nM siRNA for 24 and 48 h, respectively.
