# Supplementary material for: RNAi-Mediated Knockdown of Catalase Causes Cell Cycle Arrest in SL-1 Cells and Results in Low Survival Rate of Spodoptera litura (Fabricius)
Source: PLoS One. 2013 Mar 26;8(3):e59527. doi: 10.1371/journal.pone.0059527 (PMC3608696; doi:10.1371/journal.pone.0059527)
Supplement: Figure S4 — Increase of caspase-3 activity in SL-1 cells after treated with siRNA at different time points. Positive control: untreated with siRNA. Negative control: treated with unrelated siRNA. Data are expressed as arithmetic means ± S.E.M of three independent experiments. Treatment means sharing the same letter are not significantly different from each other (Tukey’s tests, P<0.05). (DOC) [file pone.0059527.s004.doc]

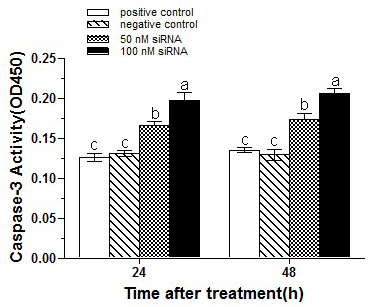


**Figure S4 Increase of caspase-3 activity in SL-1 cells after treated with siRNA at different time points.**

Positive control: untreated with siRNA. Negative control: treated with unrelated siRNA. Data are expressed as arithmetic means ± S.E.M of three independent experiments. Treatment means sharing the same letter are not significantly different from each other (Tukey’s tests, *P* < 0.05).
