# Supplementary material for: RNAi-Mediated Knockdown of Catalase Causes Cell Cycle Arrest in SL-1 Cells and Results in Low Survival Rate of Spodoptera litura (Fabricius)
Source: PLoS One. 2013 Mar 26;8(3):e59527. doi: 10.1371/journal.pone.0059527 (PMC3608696; doi:10.1371/journal.pone.0059527)
Supplement: Table S1 — Different cell cycle phases of SL-1 cells treated with siRNA. (DOC) [file pone.0059527.s005.doc]

**Table S1**

Different cell cycle phases of SL-1 cells treated with siRNA

| *Groups* | *Cell cycle phases (% DNA)* | | |
| --- | --- | --- | --- |
|  | *G1 phase* | *S phase* | *G2/M phase* |
| Control (24h) | 27.31±0.29c | 41.44±0.47a | 31.25±0.21b |
| 50 nM siRNA (24h) | 35.83±0.17b（32%↑） | 36.28±0.34a（12%↓） | 25.59±0.45b（18%↓） |
| 100 nM siRNA (24h) | 44.58±0.39a（64%↑） | 12.42±0.11b（70%↓） | 42.73±0.25a（38%↑） |
| Control (48h) | 25.89±0.48c | 40.71±0.39a | 31.87±0.35b |
| 50 nM siRNA (48h) | 37.02±0.32b（36%↑） | 36.45±0.30a（10%↓） | 26.36±0.24b（17%↓） |
| 100 nM siRNA (48h) | 47.58±0.22a（84%↑） | 7.08±0.16b（83%↓） | 43.20±0.10a（36%↑） |

*Note.* Data represent mean ± S.E.M of three replicates. Mean values followed by the same letter have no significant different (*P* < 0.05) according to Tukey’s test. Values in parentheses indicate percent increase (↑) or decrease (↓) when compared with control at the same time.
